# Development of High-Field Permanent Magnetic Circuits for NMRI/MRI and Imaging on Mice

**DOI:** 10.1155/2016/8659298

**Published:** 2016-02-29

**Authors:** Guangxin Wang, Huantong Xie, Shulian Hou, Wei Chen, Xiuhong Yang

**Affiliations:** ^1^College of Science, North China University of Science and Technology, Tangshan 063000, China; ^2^Shanghai Shining Global Science and Education Equipment Corporation, Ltd., Shanghai 201806, China; ^3^Fundamental Medical College, North China University of Science and Technology, Tangshan 063000, China

## Abstract

The high-field permanent magnetic circuits of 1.2 T and 1.5 T with novel magnetic focusing and curved-surface correction are developed. The permanent magnetic circuit comprises a magnetic yoke, main magnetic steel, nonspherical curved-surface magnetic poles, plugging magnetic steel, and side magnetic steel. In this work, a novel shimming method is proposed for the effective correction of base magnetic field (*B*_0_) inhomogeneities, which is based on passive shimming on the telescope aspheric cutting, grinding, and fine processing technology of the nonspherical curved-surface magnetic poles and active shimming adding higher-order gradient coils. Meanwhile, the magnetic resonance imaging dedicated alloy with high-saturation magnetic field induction intensity and high electrical resistivity is developed, and nonspherical curved-surface magnetic poles which are made of the dedicated alloy have very good anti-eddy-current effect. In addition, the large temperature coefficient problem of permanent magnet can be effectively controlled by using a high quality temperature controller and deuterium external locking technique. Combining our patents such as gradient coil, RF coil, and integration computer software, two kinds of small animal Micro-MRI instruments are developed, by which the high quality MRI images of mice were obtained.

## 1. Introduction

Magnetic resonance imaging (MRI) has become more and more important for clinical and basic medicine. MRI technology has many important characteristics, such as noninvasive, high soft tissue contrast, and can provide distinct anatomical information of lesions. It has significant advantage in functional imaging, especially. In addition, a lot of medical researches have to be done on animals such as rats and mice. Therefore, the research and development of small animal MRI instrument are becoming more and more urgent. It is well known that the stronger magnetic field can provide a higher signal-to-noise ratio (SNR) and images with good quality [1]. However, the optimum field strength remains unknown and depends upon many factors such as leakage magnet, image inhomogeneities, and eddy currents induced by fast switching gradients of the magnetic field gradients. At present, the mainstream of small animal MRI pieces of equipment with the magnetic field strength ≥7 T [2, 3] is generally produced on the basis of superconductor technology. Moreover, the price of these pieces of equipment is very high, and the laboratories in some universities cannot afford them. Actually, it is not necessary to perform small animals imaging by using ultra-high-field MRI instrument. For permanent magnet type MRI instrument, when the magnetic field strength is about 1.5 T, it can meet the requirements of small animal structure imaging and preliminary functional imaging.

*B*_0_ magnet is the most important part of permanent magnet type MRI instrument. Currently, the magnetic field strength of *B*_0_ magnet is generally less than 1 T. In order to increase magnetic field intensity, reduce leakage magnet, and achieve shimming, some researchers have adopted different methods, such as adding yokes [4], pole shoes [5], and shim rings [6] in *B*_0_ magnet. It is reported that *B*_0_ magnet can generally be improved by the optimization of magnetic pole shapes [7], adding adjustable shim pieces to the C-shaped magnetic poles [8], and changing magnet yokes [9]. At present, a 0.6 T MRI instrument with bore 450 mm was developed successfully [10]. The research and development of 1.0 T MRI system is also reported [11, 12]. When the magnetic field strength of cylindrical permanent magnet is up to 1.5 T, it is very difficult to install the compensation coils and solve the problem of base magnetic field uniformity. Yamada et al. [13] investigated the potential of such systems in functional MR imaging (fMRI) of somatosensory cortex activity elicited by forepaw stimulation in medetomidine-sedated rats by using a 1.5 T compact imager. Haishi et al. [14] developed a 2.0 T permanent magnetic circuit for NMR/MRI and obtained MRI image of a human finger in vivo. Moreover, the images of a live animal are not obtained in this reference. Tamada et al. [15] also developed a 2.0 T permanent magnet using a biplanar single-channel shim coil, but the mass of this kind of permanent magnet is bigger, and the space utilization of *B*_0_ magnet is lower. Therefore, the increase of *B*_0_ magnetic field in keeping large utilization efficiency of magnetic space is very important for upgrading permanent magnetic circuit for NMRI/MRI. The Magnetic Materials Research Center of Shin-Etsu Chemical Co., Ltd., has successfully developed the world's largest large-scale magnetic circuit for a Halbach type permanent magnet with a total weight of approximately 9.5 tons [16]. Although it is the largest permanent magnetic circuit generating a strong magnetic field, it will be used mainly on the production processes for MR sensor for use in MRAMs (magnetoresistance random access memories) and encoders for position detection. Moreover, our developed 1.2 T and 1.5 T permanent magnetic circuits for a half-Halbach type permanent magnet are used mainly for small animal Micro-MRI instruments.

Shimming is also a key problem in the development of permanent magnetic circuit [17–20], which consists of the passive shimming and the active shimming. The passive shimming technology is invented in the early 1990s [17]. To date, the passive shimming of permanent magnet mainly draws from one of the superconducting magnets [21]. There is no special shimming method tailored to the characteristics of permanent magnet, and the shimming mainly depends on experiences. So the shimming of permanent magnet has not made a substantial progress. At present, many optimization methods [22–29] on permanent magnet shimming are proposed, such as linear programming method [22], dynamic programming method [23], linear integer programming method [24], mixed integer programming method [25], successive approximation method [26], and other passive shimming technologies [27–29]. In order to realize the shimming of permanent magnet, the working space should be away from shim pieces, which produced an uneven local small magnetic field. Therefore, the effective working space will become small. This is also a primary factor restricting the development of permanent magnet. At present, it is reported that shimming method adding thin iron pieces is often used. Owing to the edge effect on thin iron piece shimming, the effective shimming space becomes small and the efficiency reduces. In the study, we adopted the passive shimming method of cutting, grinding, and fine processing technology in realizing the homogeneity field. The active shimming of permanent magnet is generally obtained by adding several high-order current coils. These coils may correct the complex inhomogeneity of *B*_0_ magnet. Tamada et al. [30] proposed a new planar single-channel shim coil in the magnet gap. The coil design is based on the superposition of multiple circular currents and the stream function method. In this study, the active shimming is obtained by adding higher-order current coils in the remaining spaces of RF coil and gradient coil, which not only achieves the shimming but also saves the magnetic field space.

The eddy current problem in permanent magnet is also very prominent due to the existence of more ferromagnetic materials. The eddy current will increase when ferromagnetic pieces and magnetic yokes are directly connected to permanent magnetic circuit [31–34]. At present, the active self-shielding [31–33] is a most competent method on solving the eddy current. The active self-shielding gradient coil may be obtained by installing a shielding coil outside gradient coil. When the current phases of self-shielding coil and gradient coil remain strictly the same, the eddy current of superconducting magnet may effectively be eliminated. However, it is not suitable for permanent magnet to install large active self-shielding coil because the active self-shielding coil takes up a lot of space. Making an anti-eddy-current board is currently a popular method of reducing the eddy current for permanent magnet instrument [34]. Moreover, the anti-eddy-current board will occupy valuable working space. Therefore, it is also not an ideal method of reducing eddy current.

**Figure 1:**
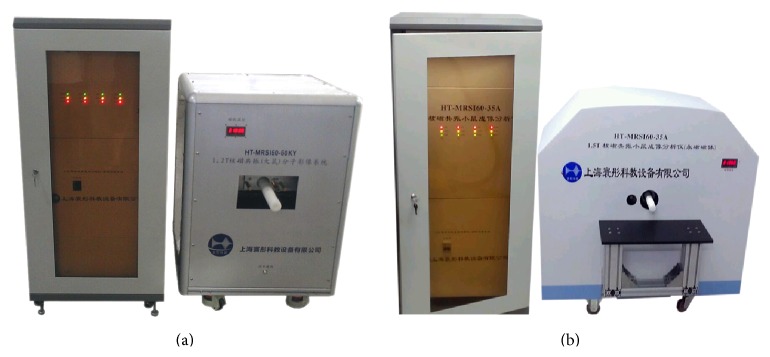
MRI systems with 1.2 T (a) and 1.5 T (b) permanent magnetic circuits.

To solve these problems, we made the improvement from three aspects according to the characteristic of permanent magnet. (1) The improvement on *B*_0_ magnet of permanent magnetic circuit: in order to increase the magnetic field strength of *B*_0_ magnet, the space efficiency, and uniformity, high-field permanent magnet system with magnetic focusing and curved-surface correction is developed. (2) The improvement on the shimming of *B*_0_ magnet: self-developed novel shimming scheme is based on passive shimming and active shimming. In the passive shimming, the traditional passive shimming method adding shim pieces is subverted, and the cutting, grinding, and fine processing technology of magnetic poles is adopted for realizing the homogeneity field. The active shimming method adding higher-order coils is proposed, which helps reduce the shimming difficult and saves the working space. (3) The improvement of the anti-eddy-current technology on *B*_0_ magnet: a new magnetic pole is formed by connecting alloy magnetic pole with main magnetic steel together. The nonspherical curved-surface magnetic poles made of the self-developed MRI dedicated alloy replaced pole pieces and anti-eddy-current board. The MRI dedicated alloy is a kind of high electrical impedance permanent magnet material, which may effectively reduce the eddy current. By using the above technologies, along with gradient coil [35], RF coil [36], and integration computer software [37], 1.2 T and 1.5 T small animal Micro-MRI instruments (Figure 1) are developed, by which the high quality MRI images of mice were obtained.

## 2. Development of Permanent MRI Instrument 

### 2.1. The Design of Permanent Magnetic Circuit

**Figure 2:**
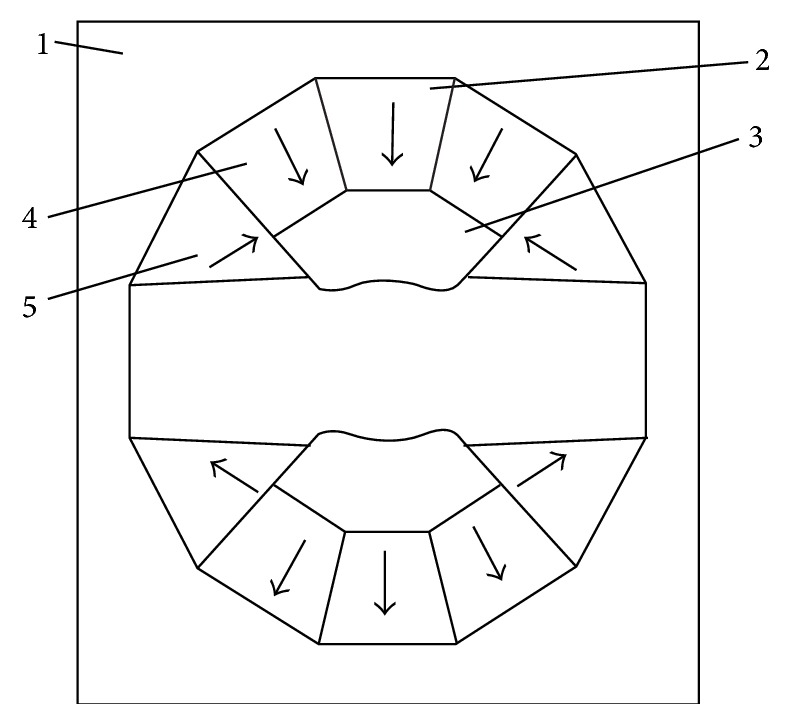
Cross-section schematic of permanent magnetic circuit. 1, magnetic yoke, 2, main magnetic steel, 3, nonspherical curved-surface magnetic poles, 4, plugging magnetic steel, and 5, side magnetic steel. The arrows represent the direction of magnetic susceptibility of magnetic steel.

**Figure 3:**
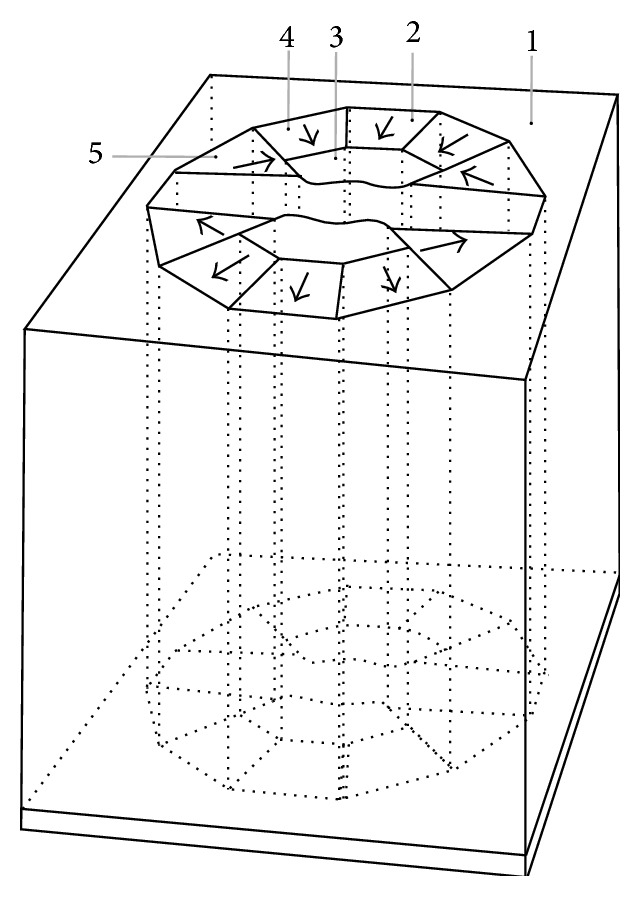
Cross-sectional stereogram of permanent magnetic circuit.

The cross-section schematic and stereogram of permanent magnet circuit are shown in Figures 2 and 3, respectively. Herein the nonspherical curved-surface magnetic poles made of the magnetic resonance imaging dedicated alloy are two saddle-type rotary curved surfaces, have the same shapes, and are symmetrically distributed at the center of the magnetic yoke. Two pieces of main magnetic steel (MMS) are, respectively, positioned behind the two magnetic poles; the plugging magnetic steel is deviated from the center position and is, respectively, positioned behind the two magnetic poles and positioned on the two side surfaces of the main magnetic steel; the side magnetic steel is, respectively, positioned on the side surfaces of the two magnetic poles and arranged on the outer side of the plugging magnetic steel. The main magnetic steel and the plugging magnetic steel are made of permanent magnet materials with high coercive force; the side magnetic steel is made of permanent magnet materials with higher coercive force; the coercive force of the side magnetic steel is over 20 percent higher than that of the main magnetic steel.

**Figure 4:**
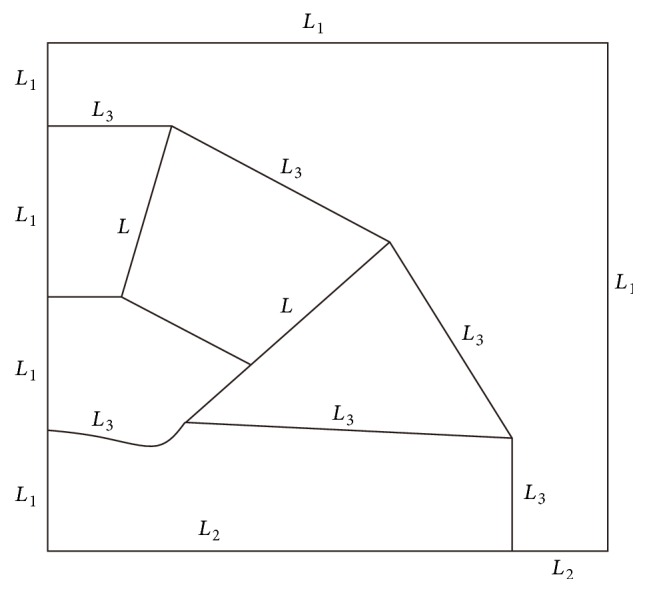
The boundary schematic of selected 1/4 permanent magnet in computation. *L*_1_ is the boundary parallel to magnetic induction lines. *L*_2_ denotes the symmetrical boundary perpendicular to magnetic induction lines. *L*_3_ denotes the border between two materials such as iron and NdFeB, air and NdFeB, magnetic poles alloy and NdFeB, and magnetic poles alloy and air. *L* is the boundary of magnetizing current.

**Figure 5:**
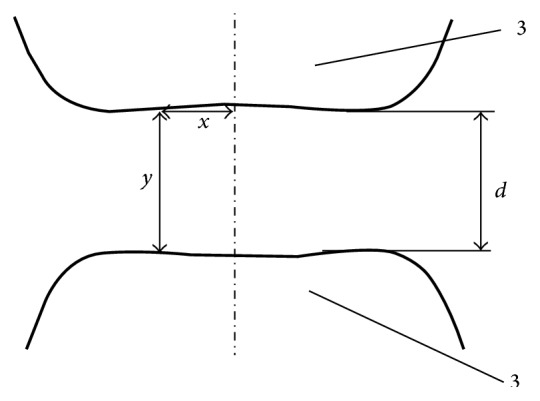
The shape and structure schematic of nonspherical curved-surface magnetic poles is demonstrated. The magnetic poles (see label 3 in Figure 2) with saddle-type rotary curved surfaces are symmetrically distributed at the center of the magnetic yoke.

The specific sizes of permanent magnetic circuit are determined by the inverse design of magnetic field distribution, and the design generally consists of three stages. First, the magnetic field distribution of permanent *B*_0_ magnet is determined within the magnetic field theory. Second, the magnetic circuit and the sizes of permanent magnet and ferromagnetic parts are determined according to magnetic properties of special alloy and NdFeB material, magnet working gap, and magnetic flux density (see Figure 4). In order to correct the error causing more simplified processing, the structure parameters of permanent *B*_0_ magnet are further optimized by means of MATLAB simulation. At last, the exact sizes of permanent magnetic circuit are determined on the basis of the experiments of magnetic field design and permanent magnetic circuit manufacture. Considering high symmetry of closed permanent magnet circuit, the sizes of two-dimensional permanent magnet circuit are determined firstly, by which the ones of three-dimensional permanent magnet circuit are deduced. The electromagnetic field equation of the simplified main magnet model is given as in [38]. We only drew boundary schematic of symmetric permanent magnetic circuits in Figures 9 and 10. *Ω*(*L*_1_ ∪ *L*_2_ ∪ *L*_3_ ∪ *L*) in (1) denotes the entire solution domain:

(1) ∂∂xv∂A∂x+∂∂yv∂A∂y=0, ΩL1∪L2∪L3∪L,

(2) A=0,L1,

(3) ∂A∂r=0,L2,

(4) v∂A∂nL3+=v∂A∂nL3−,L3,

(5) v∂A∂nL+−v∂A∂nL−=j,L,

where *A* is the undetermined magnetic vector potential. The reluctivity *v* is equal to the reciprocal of the permeability *μ*.    *μ* denotes the relative permeability of permanent magnetic material along the easy magnetization direction. The permeability *μ* is regarded as the permeability of vacuum *μ*_0_ in computation. *j* is the density of bound current. It is very difficult to obtain analytical solutions of the above equations. The sizes of magnetic poles and side magnets are obtained using the finite element method and the CUDAGPU software. The objective function of utilization coefficient on permanent magnet is given as

(6) $$\begin{array}{c} M=\frac{\int \upsilon_{c}{\left|B_{c}\right|}^{2}dV}{\int \upsilon_{m}\left|{J_{0}}^{2}\right|dV}, \end{array}$$

where *υ*_*c*_ denotes the space area between two poles of *B*_0_ magnet, *B*_*c*_ is the magnetic density, *υ*_*m*_ denotes the volume of space occupied permanent magnet, and *J*_0_ is the modulus of residual magnetization vector in permanent magnetic material. In practical design, we selected the utilization coefficient as large as possible (*M* = 0.7). The permanent magnet is made of N50-type high-grade steel (residual magnetic flux *B*_*r*_ = 1.4 T) [39]. Based on numerical calculation and manufacturing experience, the angles between main magnetic steel, nonspherical curved-surface magnetic poles, plugging magnetic steel, and side magnetic steel are determined. The sizes of each part of 1.2 T and 1.5 T permanent circuits are also determined. Their specific sizes may be seen in Figures 5, 9, and 10 and Tables 1–3.

The shape and structure schematic of nonspherical curved-surface magnetic poles is demonstrated in Figure 5. The magnetic poles (3 in Figure 2) with saddle-type rotary curved surfaces are symmetrically distributed at the center of the magnetic yoke. The closest distance *d* between two magnetic poles is the magnetic poles gap, *x* is the distance from a location point on the curved surface to the center of the magnet, and *y* is the distance between the corresponding location points of two rotating curved surfaces. In designing the curved-surface magnetic poles, the magnetic poles gap (*d*) must have higher precision at any angle. The relative error of *d* is less than 0.0003 mm (0.3 *μ*m). The two curved surfaces must have higher symmetry, and the relative error is less than 0.0003 mm (0.3 *μ*m). And the two curved surfaces must be high symmetrical on the center dotted line, whose relative error is less than 0.0005 mm (0.5 *μ*m). In Table 1, the size of magnetic poles with saddle-type rotary curved surfaces is demonstrated by using the quadratic interpolation. The data error in Table 1 is generally less than 0.1%. By the adoption of a magnetic focusing technology, high-field 1.2–2.1 T intensity can be achieved. The uniform field of a magnet is corrected by the curved surfaces, so that extremely high space efficiency and more than ten times the uniformity are achieved.

### 2.2. *B*_0_ Magnet Shimming

The highly homogeneous magnetic field is required in permanent magnet type MRI system. The basic magnetic field cannot meet the high uniformity requirement of MRI systems, so it is rather important to achieve the shimming of *B*_0_ magnet. At present, the shimming methods mainly include the active shimming and the passive shimming. The passive shimming adding ferromagnetic pieces is a popular method of achieving permanent magnet shimming. Moreover, the uneven local small magnetic field generating shim pieces seriously interferes with the basic magnetic field. In order to weaken the interference of the local small magnetic field, the working space should be away from these shim pieces. Therefore, the effective working space and the space utilization efficiency will greatly decrease. In keeping the invariable working space, the magnetic gap should become large, which will lead to the decrease of the magnetic field strength. It is well known that the utilization space of permanent magnet is proportional to the cube of *B*_0_ magnet sizes. The magnetic steel mass will greatly increase for keeping the same base magnetic field, which also increases the difficulty of manufacturing permanent magnet system. In addition, the mutual interference between ferromagnetic pieces and magnetic poles will further increase the difficulty of achieving the shimming of permanent magnet. The above factors are also the main reasons hindering the breakthrough of high-field permanent Micro-MRI instrument so far. In this study, the cutting, grinding, and polishing techniques of aspheric optical components were transplanted to the processing of magnetic poles surface and the shimming practice, by which the shimming difficulty of permanent magnet is reduced.

The good shimming is dependent on a high-precision measurement technology. In order to achieve high-accuracy shimming of permanent magnet, it is necessary to have a high-precision measurement technology. In designing permanent magnet system, the magnetic field data of 2048 collected points are measured point by point by using frequency-spectrum method, and then the processing plan is determined according to measuring magnetic field data of these points. In the practical work, measurement and high-precision processing may be carried out synchronously without the interferences of shim pieces, and the cutting, grinding, and polishing technology does not need to occupy much space. Therefore, the computation difficulty will dramatically decrease. Based on accumulated fine processing experience, the magnet poles with high-fineness surface are obtained by using the processing technology. The surface evenness of magnetic poles can be controlled in 0.3~0.1 *μ*m, and so the magnetic field homogeneity of *B*_0_ magnet will increase. The magnetic field after passive shimming needs further optimization by adding several groups of current coils on two-opposite-poles surface of magnet. The basic magnetic field is a constant field, and the scalar magnetic potential *V* satisfies Laplace equation; that is, Δ*V* = 0. The expansion equation of magnetic field *B*_*z*_ in rectangular coordinate system is given as [38]

(7) Bzx,y,z=A10+2A20+3A21x+3B21y+32A302z2−x2−y2+12A31zx+12B31zy+15A32x2−y2+30B32xy+4A40zz2−23x2+y2+152A41x4z2−x2−y2+152B41y4z2−x2−y2+···.

Generally, only *B*_*z*_ is computed in the shimming. The first term in (7) is a uniformity field value preserved in shimming. The nonuniformity parts of the base magnetic field after passive shimming have usually a certain gradient. The second, third, and fourth terms denote linear gradients along *x*, *y*, and *z* coordinate axes, respectively. The fifth, sixth, seventh, and eighth terms are second-order nonuniformity parts. The ninth, tenth, eleventh, and twelfth terms are third-order nonuniformity parts. The developed high-order gradient coils still retain the early MRI technique characteristic of compensating the gradient of base magnetic field. When the magnetic field induced by these high-order gradient coils exactly offsets the gradient of main magnet itself in a specific direction, these high-order gradient coils are regarded as the shimming coils. In addition, when the resonance frequencies of these points in the high uniform magnetic field tend to be the same, the vibration amplitude of every point is the biggest, by which 27 groups of higher-order shimming coils including *x*, *y*, *z*, *xy*, *yz*, *xz*, *x* ^2^ − *y* ^2^, *R* ^2^, *R* ^3^, *Rz* ^2^, (*x* ^2^ − *y* ^2^)*z*, *y* ^3^, *xyz*, and other third-order parts are preliminary designed. The shimming method adding high-order current coils is named as the active shimming. In the study, a novel shimming scheme is based on passive shimming and active shimming. In addition, we took a year to track the imaging of small animal permanent magnet type MRI instrument and fulfill correction of magnetic poles continuously. A good shimming of permanent magnet is obtained when the high-order shimming coils reduce to 9 groups.

### 2.3. Eddy Current of Permanent Magnet

**Figure 6:**
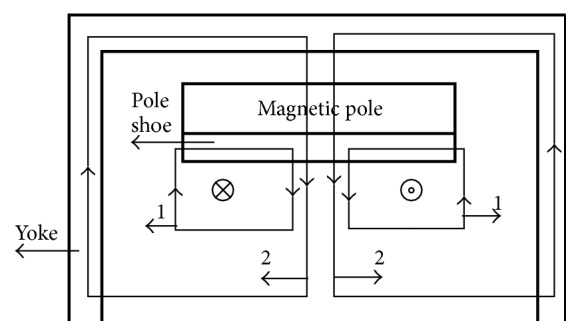
Magnetic circuits with traditional gradient coils.

**Figure 7:**
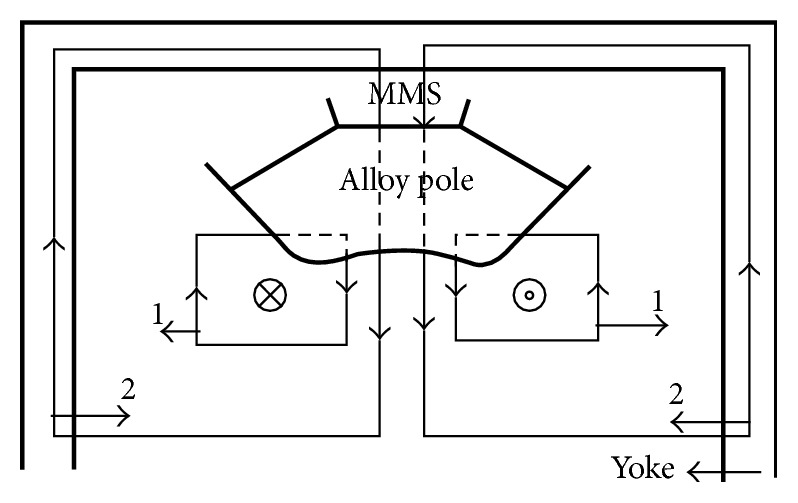
High-impedance special alloy magnetic poles without pole shoes. The magnetic circuit may block the gradient pulse and prevent the eddy currents.

In the conventional permanent magnetic circuit, two possible magnetic flux routes generating gradient magnetic field are shown in Figure 6. Although the pole shoes are made from high permeability and impedance material, it is also very difficult for permanent magnetic poles to prevent the closed gradient magnetic flux passing through magnetic yokes. At present, adding an anti-eddy-current board between gradient coils and magnetic poles is the most mainstream and effective method of eliminating the eddy current in permanent magnetic circuit. However, the anti-eddy-current board will occupy large working space. It is also not an ideal method of reducing eddy current. In order to eliminate the eddy current, we replaced pole pieces and anti-eddy-current board with an alloy magnetic pole. These magnetic induction lines of magnetic circuits 1 and 2 in Figure 7 interrupt in nonspherical curved-surface alloy magnetic poles, which may effectively prevent generation of eddy current. Considering the symmetry of the magnetic circuit, the upper part of the magnetic circuit schematic diagram was given in Figures 6 and 7.

The medical magnetic resonance imaging system can be regarded as the magnetic field stacking a rapidly changed gradient magnetic field based on the uniform magnetic field. The system consists of *X*, *Y*, *Z* three groups of gradients. The spatial information is determined by changing the sizes of *X*, *Y*, *Z* gradients. So the accuracy and switching speed of the gradient magnetic field determine the imaging quality. The main factor affecting the accuracy and switching speed of the gradient magnetic field is the eddy current inducing the nearest magnetic poles extreme. The eddy current is a result of the interaction between the magnetic field and the current inducing the conductor in the variable magnetic field. Adding the active self-shielding coils is an effective method of reducing the eddy currents in superconducting magnetic circuit to date. Moreover, it is not very ideal for permanent magnetic circuit to add the active self-shielding coils. The active self-shield coils will take up too much space and need to pay a high price [31]. At present, making magnetic poles with the amorphous magnetic material, sheet lamination and silicon steel splicing are the popular solution methods of eliminating the eddy current. The above schemes can only be used below the 1.0 T magnetic resonance system owing to the low saturation magnetic intensity. In order to eliminate the eddy currents, the magnetic resonance imaging dedicated alloy with high-saturation magnetic field induction intensity and high electrical resistivity is developed, and nonspherical curved-surface magnetic poles made of the dedicated alloy replaced traditional magnetic poles and pole pieces which are easy to induce the eddy currents. A preparation method of the magnetic resonance imaging dedicated alloy is given as follows. The dedicated alloy is Fe-Co-Al fe-co-based alloy, the mass percent of the Co content is 5%–35%, the mass percent of Al content is 2%–6%, the mass percent of Si content is 0.5%–1%, the mass percent of Mo content is 2%-3%, required impurities include C, P, S, and Ni which are smaller than 0.01%, and the rest is Fe. The electrical resistivity of the alloy is larger than 50 *∗* 10^−8^  Ω/m, the saturation flux density is larger than 1.4–2.3 T, the initial permeability is larger than 2000, the magnetic permeability at 1.0–1.5 T is larger than 10000, and preparation is performed by a vacuum metallurgy method. According to the magnetic resonance imaging dedicated alloy and the preparation method, high-saturation magnetic field induction intensity and high electrical resistivity can be achieved, the alloy is a magnetic pole material that is used for 1.0 T–2.1 T permanent magnet magnetic resonance imager, and the anti-eddy effect is good.

### 2.4. Gradient Coil, RF Coil, and Temperature Control

**Figure 8:**
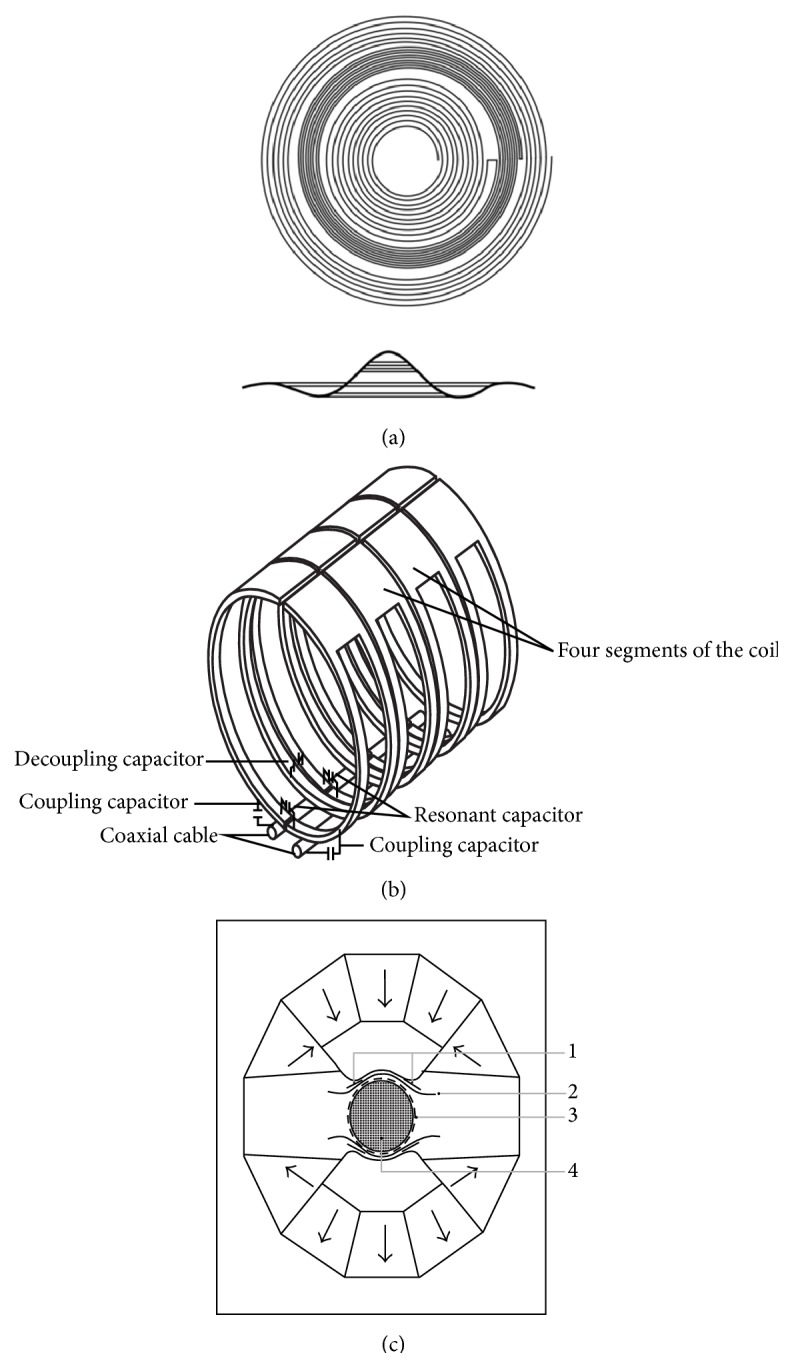
(a) Gradient coil. (b) RF coil. (c) The optimal allocation of all kinds of coil. 1, shimming coil. 2, gradient coil. 3, RF coil. 4, DSV.

We developed a self-shielding gradient coil with straw-hat curved-surface shape. The self-shielding gradient coil with high linearity, high efficiency, and small size is not only different from cylindrical superconducting gradient coil, but also different from the planar gradient coils. The eddy currents are effectively minimized by installing three groups of high-order gradient coils in small animal permanent MRI system at 0.5 T [40]. When the main magnetic field strength is up to 1.5 T [41], the stronger eddy current which worsens the linearity of the gradient magnetic field cannot effectively be eliminated by only adding self-shielding gradient coils. In addition, the magnetic resonance imaging dedicated alloy replaced traditional magnetic poles and pole pieces which are easy to induce the eddy currents. The self-shielding gradient coil picture is shown in Figure 8(a). Self-developed RF coil not only absorbs the advantage in which the saddle-shaped coil can provide the uniform RF field in the vertical direction of *B*_0_ magnetic field, but also absorbs the advantage of the solenoid-shaped coil with high sensitivity and uniformity field. The single-channel integral coil formed orthogonal transmitter coil and receiver coil avoids the complex array coil circuit. The single-channel integral coil has a faster imaging speed owing to selecting higher magnetic field strength and superior gradient coils. The RF coil picture is also shown in Figure 8(b). For improving the space utilization of magnetic poles gap and reducing the volume of *B*_0_ magnet, all kinds of coils were arranged scientifically in Figure 8(c). Taking the curved type gradient coils, for example, three groups of coils were placed in the remaining gaps between the uniform field coils. This design not only obtained the self-shielding gradient coil, but also did not take up too much space.

The spatial distribution of the magnetic field may also vary with temperature. The stability of *B*_0_ magnetic field determines the image quality. Therefore, the frequency or current compensation is an effective method of obtaining the stable magnetic field. Haishi et al. [42] developed a 1.0 T MR microscope using a NdFeB permanent magnet, and they developed an internal NMR locking technique or the imaging sequences because the magnetic field of the permanent magnet had a large temperature coefficient (−1200 ppm/deg.). In the study, the temperature drift can be effectively controlled by using a high quality temperature controller and “deuterium external locking” technique. The “deuterium external locking” technique is a method of compensating the magnetic field drift by the additional magnetic field inducing the current of the lock field coil, and the current of the lock field coil is adjusted by detecting the deuterium signal. The temperature drift is controlled around 1 ppm in 10 minutes (2~3 ppm/h) in imaging experiments.

## 3. Discussion

**Figure 9:**
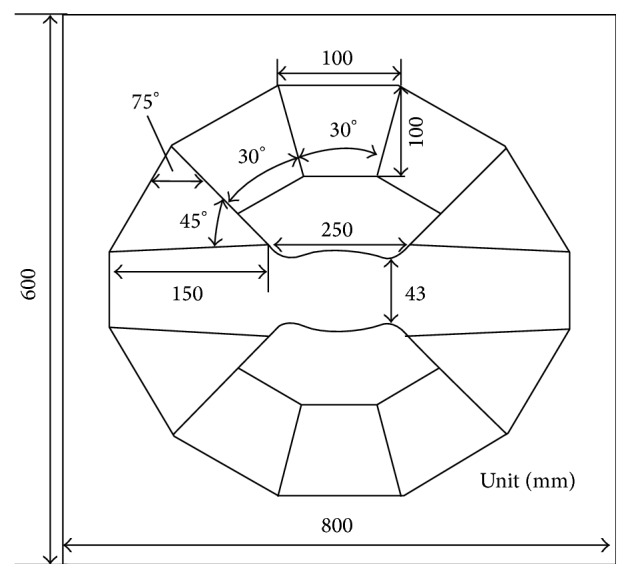
The size of each part of 1.5 T permanent magnetic circuit.

**Figure 10:**
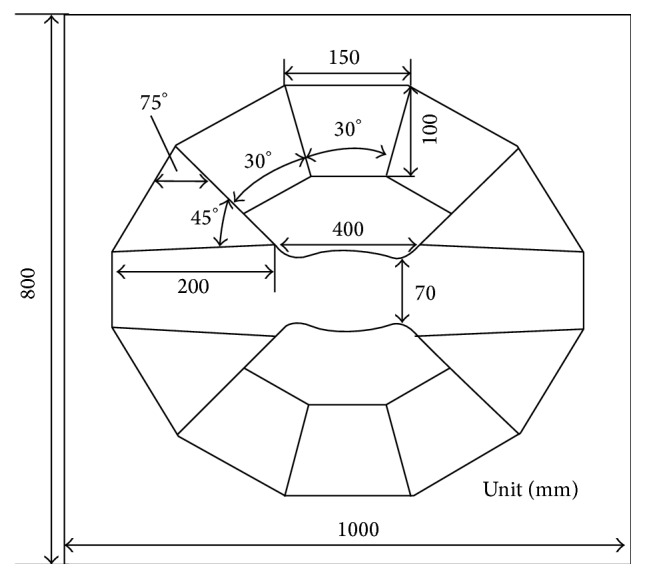
The size of each part of 1.2 T permanent magnetic circuit.

As the experiment, two kinds of permanent magnetic circuits are developed. One magnetic circuit is with a main magnetic field strength 1.5 T, a magnetic gap 43 mm, a cylinder shimming space with 35 mm (diameter) × 60 mm (height), and a space utilization efficiency of 81% (the space utilization efficiency is the ratio of the volume of the working space to the volume between magnetic poles); the static homogeneity is 1 ppm homogeneity over 20 mm DSV. According to the data in Table 2, the specific dimensions of the 1.5 T permanent magnetic circuit are given in Figure 9. The other one is with a main magnetic field strength 1.2 T, a magnetic gap 70 mm, a cylinder shimming space with 60 mm (diameter) × 100 mm (height), and a space utilization efficiency of 85%; the static homogeneity is 1 ppm homogeneity over 20 mm DSV. According to the data in Table 3, the specific dimensions of the 1.2 T permanent magnetic circuit are given in Figure 10. 1.2 T and 1.5 T permanent magnetic circuits, gradient coils, RF coils, and integrated software systems with independent intellectual property rights are assembled into 1.2 T and 1.5 T MRI instruments. The high quality images of mice are obtained by using self-developed two kinds of Micro-MRI instruments. The 1.2 T and 1.5 T Micro-MRI instruments are demonstrated in Figure 1.

In order to implement mice imaging, 12 healthy male mice with the age of 4 weeks were obtained from Shanghai Experimental Animal Center. Their masses are between 17 and 20 grams. With the approval of the Ethical Committee of North China University of Science and Technology, the experiment of live mice and the injection of carcinogenic urethane to live mice were performed. The dosage of urethane anesthesia injecting into the abdomen of the healthy male mice is 1 g/kg. It took 4 minutes to 10 minutes for one 3D-T1 data set of one mouse under the anesthesia. The imaging scans of mice were performed after 5 minutes. The coronal images of a live mouse are obtained by using the 1.2 T small animal MRI instrument. The cross-sectional images of a dead mouse are obtained by using the 1.5 T small animal MRI instrument.

**Figure 11:**
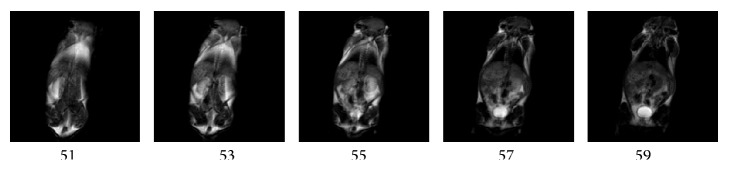
Coronal images of the 51st, 53rd, 55th, 57th, and 59th layer slices on live mice are demonstrated from left to right. Parameters of MRI at 1.2 T: TR/TE = 100 ms/15 ms, cylindrical shimming volume 60 mm × 100 mm, FOV = 60 mm × 100 mm, slice thickness 0.3 mm, data matrix 512 × 36 × 256, and imaging matrix 1024 × 1024.

**Figure 12:**
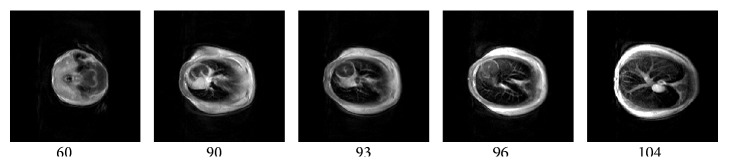
Cross-sectional images of the 60th, 90th, 93rd, 96th, and 104th layer slices on a dead mouse are demonstrated from left to right. Parameters of MRI at 1.5 T: TR/TE = 200 ms/15 ms, cylindrical shimming volume 35 mm × 60 mm, FOV = 35 mm × 60 mm, slice thickness 0.4 mm, data matrix 32 × 512 × 256, and imaging matrix 512 × 512.

The SE sequence and T1-weighted images of mice are obtained by using self-developed 1.2 T and 1.5 T small animal MRI instruments. *x* and *y* direction phase codes, *z* direction frequency code, and sinc form RF pulse are selected in coronal and cross-sectional scans of several mice. The 128 layers images of a live mouse are obtained in every imaging experiment. The imaging parameters of 1.2 T and 1.5 T MRI instruments are set as follows, respectively. (1) Coronal imaging parameters on the 1.2 T MRI instrument: TR/TE = 100 ms/15 ms, cylindrical shimming volume is 60 mm × 100 mm, the field of view (FOV) = 60 mm × 100 mm, slice thickness is 0.3 mm, data matrix is 512 × 36 × 256, and imaging matrix is 1024 × 1024. The 51st, 53rd, 55th, 57th, and 59th layer coronal images of a live mouse were demonstrated in Figure 11. These randomly selected coronal images of mice from head to tail can be clearly observed. (2) Cross-sectional imaging parameters on the 1.5 T MRI instrument: TR/TE = 200 ms/15 ms, cylindrical shimming volume is 35 mm × 60 mm, FOV = 35 mm × 60 mm, slice thickness is 0.4 mm, data matrix is 32 × 512 × 256, and imaging matrix is 512 × 512. The randomly selected 60th, 90th, 93rd, 96th, and 104th layer cross-sectional images of a dead mouse were demonstrated in Figure 12. The internal organs of the dead mouse can be clearly observed in Figure 12. The results show that higher field intensity of permanent magnet may improve greatly the imaging quality of small animals.

## 4. Conclusions

In the study, we developed a magnetic resonance imaging dedicated alloy with high-saturation magnetic field induction intensity and high electrical resistivity, and high-field permanent magnetic circuits of 1.2 T and 1.5 T with novel magnetic focusing and curved-surface correction. The nonspherical curved-surface magnetic poles made of the dedicated alloys are two saddle-type rotary curved surfaces, have the same shapes, and are symmetrically distributed at the center of the magnetic yoke. A new magnetic pole is formed by connecting the nonspherical curved-surface alloy magnetic pole with main magnetic steel together, which replaced traditional magnetic pole and poles pieces easily induced the eddy currents. The plugging magnetic steel is deviated from the center position and is, respectively, positioned behind the two magnetic poles and positioned on the two side surfaces of the main magnetic steel; the side magnetic steel is, respectively, positioned on the side surfaces of the two magnetic poles and arranged on the outer side of the plugging magnetic steel; the main magnetic steel and the plugging magnetic steel are made of permanent magnet materials with high coercive force; the side magnetic steel is made of permanent magnet materials with higher coercive force; the coercive force of the side magnetic steel is over 20 percent higher than that of the main magnetic steel. By the adoption of a magnetic focusing technology, high-field 1.2–2.1 T intensity can be achieved, and the uniform field of a magnet is corrected by the curved surfaces. In terms of shimming, the conventional passive shimming field method adding shim pieces is abandoned, and a telescope aspheric cutting, grinding, and fine processing technology of the nonspherical curved-surface magnetic poles is used. Meanwhile, the active shimming technology adding higher-order gradient coils is adopted, which effectively corrects the uniform field of *B*_0_ magnet. Based on accumulated fine processing experience, the magnet poles with high-fineness surface are obtained by using the telescope aspheric cutting, grinding, and fine processing technology. The surface evenness of magnetic poles can be controlled in 0.3~0.1 *μ*m. After adding third-order shimming coils, the static homogeneity is 1 ppm homogeneity over 20 mm DSV. In addition, the large temperature coefficient problem of permanent magnet can be effectively controlled by using a good temperature controller and “deuterium external locking” technique; the new magnetic poles and gradient coils are optimally designed in terms of shape and structure. The shimming coils occupy the space between gradient coil and concave magnetic poles. Therefore, all the parts are optimally arranged and occupy the smallest magnetic field space. Combining our patents such as gradient coil, RF coil, and integration computer software, 1.2 T and 1.5 T small animal Micro-MRI instruments are developed, by which the high quality coronal and cross-sectional images of mice are obtained. Therefore, it is very necessary to strengthen the study of permanent magnet system for updating small animal MRI instruments.

## Figures and Tables

**Table 1:** The size of magnetic poles with saddle-type rotary curved surfaces. *d* is the magnetic poles gap, *x* is the distance from a location point on the curved surface to the center of the magnet, and *y* is the distance between the corresponding location points of two rotating curved surfaces.

*x*/*d*	0.00	0.4	0.8	1.2	1.6	2.0	2.4	2.8	3.2	3.24	3.28	3.32	3.36	3.4	3.52
*y*/*d*	1.08	1.076	1.063	1.042	1.014	1.0	1.0	1.023	1.18	1.21	1.24	1.27	1.30	1.34	1.46

**Table 2:** The size of permanent magnetic circuit with the magnetic field strength 1.5 T. *x* is the distance from a location point on the curved surface to the center of the magnet. *y* is the distance between the corresponding location points of two rotating curved surfaces. The units of *x* and *y* are mm.

*x*	0	12	24	36	48	60	72	84	96	97.2	98.4	99.6	100.8	102
*y*	43.2	43.04	42.54	41.69	40.56	40.0	40.0	40.93	47.38	48.41	49.53	50.74	52.05	53.46

**Table 3:** The size of permanent magnetic circuit with the magnetic field strength 1.2 T. *x* is the distance from a location point on the curved surface to the center of the magnet. *y* is the distance between the corresponding location points of two rotating curved surfaces. The units of *x* and *y* are mm.

*x*	0	28	56	84	112	140	168	196	224	226.8	229.6	232.4	235.2	238
*y*	75.6	75.32	74.44	72.95	70.98	70.0	70.0	71.63	82.91	84.72	86.67	88.80	91.08	93.55
